# Predictive Computer Models for Biofilm Detachment Properties in *Pseudomonas aeruginosa*

**DOI:** 10.1128/mBio.00815-16

**Published:** 2016-06-14

**Authors:** Nick G. Cogan, Janette M. Harro, Paul Stoodley, Mark E. Shirtliff

**Affiliations:** a Department of Mathematics, Florida State University, Tallahassee, Florida, USA; b Department of Microbial Pathogenesis, School of Dentistry, University of Maryland—Baltimore, Baltimore, Maryland, USA; c Departments of Microbial Infection and Immunity, Orthopedics, Center for Microbial Interface Biology, The Ohio State University, Columbus, Ohio, USA; d National Centre for Advanced Tribology at Southampton (nCATS), University of Southampton, Southampton, United Kingdom; e Department of Microbiology and Immunology, School of Medicine, University of Maryland—Baltimore, Baltimore, Maryland, USA

## Abstract

Microbial biofilm communities are protected against environmental extremes or clearance by antimicrobial agents or the host immune response. They also serve as a site from which microbial populations search for new niches by dispersion via single planktonic cells or by detachment by protected biofilm aggregates that, until recently, were thought to become single cells ready for attachment. Mathematically modeling these events has provided investigators with testable hypotheses for further study. Such was the case in the recent article by Kragh et al. (K. N. Kragh, J. B. Hutchison, G. Melaugh, C. Rodesney, A. E. Roberts, Y. Irie, P. Ø. Jensen, S. P. Diggle, R. J. Allen, V. Gordon, and T. Bjarnsholt, mBio 7:e00237-16, 2016, http://dx.doi.org/10.1128/mBio.00237-16), in which investigators were able to identify the differential competitive advantage of biofilm aggregates to directly attach to surfaces compared to the single-celled planktonic populations. Therefore, as we delve deeper into the properties of the biofilm mode of growth, not only do we need to understand the complexity of biofilms, but we must also account for the properties of the dispersed and detached populations and their effect on reseeding.

## COMMENTARY

Following the rediscovery and coining of the “term” biofilms in the late 1970s by Costerton et al. (1) from the long forgotten observance of dental plaque by Anton Van Leewenhook (2), the defining characteristics that have described biofilms have evolved. Initially biofilms were seen as uniform layers of microbes, almost like accretions of scum or dirt, with a lackluster or undistinguished phenotype. However, the truly complex nature of biofilm development was discovered with the advent of confocal microscopy. This tool allowed researchers to make the phenotypic distinction between initial attachment, maturing, and fully mature biofilm stages, as well as observe the phenomena of dispersal via individual cells or detachment of large clusters of cells, termed “floccules” (3) or “aggregates.” The early work focused on detachment due largely to physical forces, such as fluid shear or particle abrasion (4). However, it has more recently been recognized that detachment can also occur as an actively controlled process (5–8). Determination of dispersal and detachment mechanisms was quickly seized upon by biofilm researchers as a possible way to control and remove biofilms of industrial and clinical concern.

Advancements in genetic and biochemical tools facilitated further discoveries of the biofilm life cycle. Gene knockouts and reporters, antimicrobial tolerance determination, and subsequent “-omic” studies highlighted not only the unique nature of the biofilm mode of growth but also the phenotypic distinctiveness of the individual stages of biofilm development. Nutrient and microbial heterogeneity, quorum sensing systems, and properties of the host or microenvironment all added to the intractability in understanding the biofilm phenotype. The realization of the extent of the complexity meant that these microbial communities could no longer be only defined as simply bacteria attached to a hydrated surface embedded in slime. Therefore, Donlan and Costerton proposed an all-encompassing definition of biofilms in 2002 (9). They state that a biofilm is a “microbially derived sessile community characterized by cells that are irreversibly attached to a substratum or interface or to each other, are embedded in a matrix of extracellular polymeric substances that they have produced, and exhibit an altered phenotype with respect to growth rate and gene transcription” (9).

Included in this definition is the observance that bacteria can exist as detached conglomerates of cells attached to one another in “aggregates” or “floccules” (3). Although many biofilm researchers studied the detached bacterial populations, it was generally understood that cells would be released into a planktonic mode of growth from their extracellular polymeric substance (EPS) prison to reseed as single cells and start the biofilm development process anew. That is until now. The article by Kragh et al. demonstrates the importance of the relative proportions of numbers of cells in aggregates compared to those released as single daughter cells (10). Shed aggregates of cells and shed single cells each have their advantages. While motile single cells can actively locomote to escape challenging local environments for new niches, they are more susceptible to antibiotics, phagocytosis, and chemical challenges. In contrast, cells detached as aggregates have little control over their trajectory and are carried by fluid forces but have the advantage in that they remain in the protected biofilm phenotype, so when they attach to a fresh surface, they have a head start on biofilm formation (11).

This group, led by Dr. Thomas Bjarnsholt in the Costerton Biofilm Center (CBC), which is headed by a leader in the field of biofilm research, Dr. Michael Givskov, has successfully challenged a number of biofilm paradigms by demonstrating biofilm formation independent of surface attachment. His group has also been the first to challenge the *in vivo* relevance of the large and luxuriant *in vitro* biofilms with diameters approaching hundreds of micrometers and the associated “mushroom-shaped structures.” Instead, the microbes often exist as smaller cell aggregates even after attaining full maturity within affected tissues that are able to escape biopsy sampling, thereby making diagnosis of these *in vivo* biofilm infections exceedingly difficult. Their work, along with that of many others in the field, has led to recent clinical guidelines for the diagnosis of biofilm infections (12).

Yet the remarkable part of this story is that the investigators were led to perform the experiments not by a scientific hunch but instead were directed by mathematical modeling of biofilm formation. During the early years of biofilm research, the study of microbial communities had a strong foundation in engineering applications. Therefore, it is not surprising that soon after the discovery of biofilms, attempts to mathematically model their behavior quickly followed. One of the earliest successful attempts was performed at the precursor group that evolved into the NSF-ERC Center for Biofilm Engineering (13). This group modeled steady-state *Pseudomonas aeruginosa* and *Klebsiella pneumoniae* as model biofilm formers and found that the growth kinetics, glucose metabolism, and biofilm EPS production were no different from those of planktonic cells in a suspended continuous culture. However, this study was performed prior to the recognition of the complex lifestyle of biofilms and their development.

Since then, the mathematical modeling of biofilm dynamics has a relatively long history, with both continuum and discrete models being used. Kragh et al. use a discrete model to study the effect of competition between aggregates of bacteria and individual bacteria. Nicely complementing the numerical studies with experimental investigations, the study helps confirm several widely known behaviors, including growth instabilities and nutrient competition within established biofilm colonies. However, the study also probes a subtler question about the initiation of the biofilm colony. The standard, surface, growth instability in a model is typically analyzed *in silico* by stochastically “seeding” an initial colony that may be flat for a continuum model or grown by a random initial seeding on the surface for a discrete model. The differences in the dynamics between this type of instability and that in which the initial seeding combines aggregates and individuals have not been adequately characterized.

The results of the simulations, measured by the ratio of the produced daughter cells to the initial seeded cells, indicate that the aggregation plays a distinct role in the growth dynamics, especially when nutrients are scarce. This connects with previous observations from other investigators (14–16) that show a correlation between surface roughness and nutrient availability. This work also adds to the understanding of these bacterial aggregates described by other approaches that have shown that reproduction (or growth rate at least) is lower overall in larger, denser aggregates even if the change in volume is not (17). Therefore, the authors succeed in demonstrating that when studies focus on the initial stages of biofilm formation, there are circumstances in which the distinction between aggregates and individual cells must be incorporated. They also demonstrate once again the ability of mathematical models to predict and complement experimental studies.

**FIG 1:**
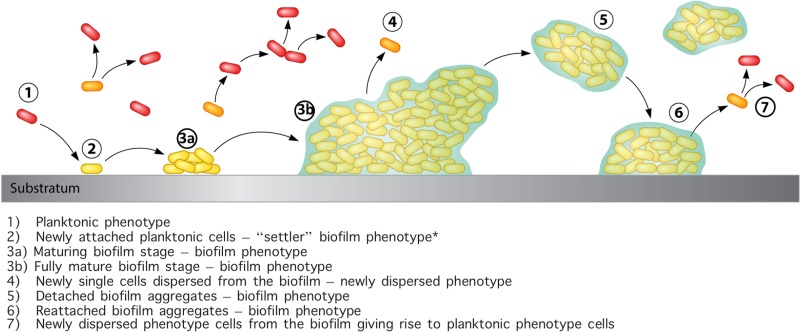
Biofilm dispersion and detachment. (Step 1) Planktonic bacteria (red single cells) can initially seed and (step 2) attach to a substratum and quickly develop into a settler phenotype (yellow single cells) and then develop to (step 3a) a maturing then (step 3b) fully mature biofilm phenotype embedded within a host and/or microbe-derived hydrated matrix. Bacteria can then spread through (step 4) single dispersed cells (orange single cells) with a unique phenotype compared to the purely planktonic mode of growth (red single cells). (Step 5) In addition, large aggregates may detach from the biofilm in a protected population that can (step 6) directly seed other surfaces or (step 7) give rise to single detached cells that subsequently develop into planktonic phenotype cells for reseeding. (It should be noted [as indicated by the asterisk] that although all biofilm bacteria in this rendering are given a similar designation, the phenotypic differences between the various stages of biofilm formation are significant and well described.)

The data from this study and others suggest that the biofilm ecosystem shows greater complexity than the binary “biofilm” or “planktonic” phenotypes would suggest. It is known that the biofilm phase has multiple phenotypes due to the development of gradients, but the new research shows that the liquid “planktonic” phase may also contain multiple phenotypes. These include large numbers of cells as detached biofilm aggregates, dispersed biofilm single cells, and the conventional planktonic cells that have originated from growth while in the planktonic phase. In addition, data suggest newly attached planktonic “pioneer” cells have a distinct phenotype as they transition into a surface-attached lifestyle. The exchange and division of cells between the attached biofilm phase and the suspended planktonic phase illustrate the need for studying biofilms not only as a whole but also at the single-cell or subpopulation level (Fig. 1).

Therefore, even though we are now 25 years on from the revelations afforded by confocal microscopy, new techniques and approaches continue to reveal new and exciting behaviors in biofilm, further adding to their recognized complexity. These discoveries come first from careful observation in laboratory models or clinical samples, followed by quantification and then mechanistic interrogation by molecular or computational methods. The multifaceted nature of these microbial social behaviors cannot, as yet, be predicted from knowledge of the genome. As new exciting methods of experimentation and imaging are developed and the imagination of the next generation of biofilm scientists is unleashed on the ever-growing problem of biofilms, more groundbreaking discoveries will surely follow.
